# High resolution melting analysis for the detection of EMS induced mutations in wheat *SbeIIa *genes

**DOI:** 10.1186/1471-2229-11-156

**Published:** 2011-11-10

**Authors:** Ermelinda Botticella, Francesco Sestili, Antonio Hernandez-Lopez, Andrew Phillips, Domenico Lafiandra

**Affiliations:** 1 Department of Agriculture, Forests, Nature and Energy, University of Tuscia, 01100 Viterbo, Italy; 2 Plant Science Department, Rothamsted Research, Harpenden, AL5 2JQ, UK

## Abstract

**Background:**

Manipulation of the amylose-amylopectin ratio in cereal starch has been identified as a major target for the production of starches with novel functional properties. In wheat, silencing of starch branching enzyme genes by a transgenic approach reportedly caused an increase of amylose content up to 70% of total starch, exhibiting novel and interesting nutritional characteristics.

In this work, the functionality of starch branching enzyme IIa (SBEIIa) has been targeted in bread wheat by TILLING. An EMS-mutagenised wheat population has been screened using High Resolution Melting of PCR products to identify functional SNPs in the three homoeologous genes encoding the target enzyme in the hexaploid genome.

**Results:**

This analysis resulted in the identification of 56, 14 and 53 new allelic variants respectively for *SBEIIa-A*, *SBEIIa-B *and *SBEIIa-D*. The effects of the mutations on protein structure and functionality were evaluated by a bioinformatic approach. Two putative null alleles containing non-sense or splice site mutations were identified for each of the three homoeologous *SBEIIa *genes; qRT-PCR analysis showed a significant decrease of their gene expression and resulted in increased amylose content. Pyramiding of different single null homoeologous allowed to isolate double null mutants showing an increase of amylose content up to 21% compared to the control.

**Conclusion:**

TILLING has successfully been used to generate novel alleles for *SBEIIa *genes known to control amylose content in wheat. Single and double null *SBEIIa *genotypes have been found to show a significant increase in amylose content.

## Background

Reserve starch represents the main component of wheat flour constituting roughly 60-70% of the wheat kernel and is chemically composed of a mixture of two glucan polymers known as amylose and amylopectin, representing 20-30% and 80-70% of total starch, respectively. The two glucan polymers differ in their degree of polymerization and of branching: amylose is essentially linear (DP < 10^4^) and amylopectin is highly branched (DP 10^5^-10^6^). The two glucan polymers contribute differently to the functional properties of starch and the modulation of amylose/amylopectin ratio has been identified as a major target in order to develop starches with novel physical-chemical properties. In particular, high amylose starch is more and more in demand because of its unique nutritional properties and also for its technological characteristics that are opening new applications both in food as well as in non-food sectors [1-5].

Nutritionists and food industries are paying increasing attention to cereals with high amylose starch as derived foods have an increased amount of resistant starch, which has a role similar to dietary fibre inside the intestine, protecting against important diet related diseases [4]. An increased knowledge of starch biosynthesis is a necessary prerequisite for the determination of effective approaches to modify the amount of amylose in starch. Several starch enzymes have been identified as key factors in the modulation of the amylose/amylopectin ratio.

The two starch polymers are synthesized from a common substrate, ADP-glucose, by different pathways. Amylose biosynthesis involves a single enzyme, GBSSI (granule bound starch synthase I), known as waxy protein. In contrast, the branched structure of amylopectin is the result of a more complex biosynthetic mechanism involving several classes of enzymes: different types of starch synthases (SSs) promote the elongation of glucan chains by catalyzing the formation of α-1,4 glucosidic bonds; starch branching enzymes (SBEs) introduce α-1,6 links into the glucan backbone; debranching enzymes (DBEs) remove excess branches from glucan chains contributing to optimal packing of the semi-crystalline structure of the starch granule [6,7].

Approaches to manipulate starch composition in wheat have involved both classical and biotechnological strategies. The silencing of genes encoding SSIIa (also known as Starch Granule Protein-1, SGP-1) and SBEIIa are currently two successful strategies for increasing amylose content. As starch granule proteins are easily detected by sodium dodecyl sulphate-polyacrylamide gel electrophoresis (SDS-PAGE), it has been possible to identify several mutant lines missing one of the three possible SGP-1 isoforms by screening natural germplasm and mutant populations [8,9]. The absence of SSIIa has been found to cause a significant increase in amylose both in bread [10] (up to 35%) and durum wheat [11] (up to 45%). In wheat two classes of SBE, SBEI and SBEII, exist; the latter comprises two isoforms, SBEIIa and SBEIIb. The loss of SBEI has been reported not noticeably to affect starch composition [12]. *SBEIIa *and *SBEIIb *genes have been characterized and found to be located on the long arm of the homoeologous group 2 chromosomes [13]. *SBEIIa *has been shown to be the most abundant isoform and is found mainly in the soluble fraction of endosperm extracts, while SBEIIb is more highly represented in starch granules [14].

The ability to silence all copies of targeted genes through the use of RNA interference (RNAi) has permitted the elucidation of the role and functionality of the two different SBEII isoforms. Silencing of the *SBEIIa *and *SBEIIb *homoeologous gene families in bread wheat showed that only the loss of SBEIIa isoform was associated with a highly increased proportion of amylose in the transgenic lines (up to 70% of total starch) [15]. Although RNAi has now been shown to be effective in the production of high amylose lines in both bread and durum wheat [15,16], the application of transgenic technology to crop improvement is still not completely accepted, encountering resistance from the general public and from governments.

Classical mutagenesis has been widely used in crop breeding over the past 60 years and is lately re-emerging as an efficient alternative to exploit and modify functionality of genes controlling important traits in crops. Chemical mutagenic treatment provides an efficient tool to generate high density mutations in the genome of the target organism, although in polyploids the presence of multiple copies of a gene has represented a major limitation in the detection of interesting phenotypes for valuable traits by forward genetics approaches. However, recent developments in sequence-level detection of mutations, coupled with the increased availability of both genomic and EST sequence data, have resulted in the development of a novel strategy of reverse genetics known as TILLING (Targeting Induced Local Lesions In Genomes) [17]. This technology was developed in Arabidopsis but has now been successfully applied to several crop species, including wheat, in which traits related to starch properties have been successfully targeted. Slade *et al. *[18] identified a total of 246 novel *waxy *(GBSSI) alleles in durum and bread wheat and crossed null mutants in different homoeologues to produce a waxy phenotype. Similarly, Sestili *et al. *[9] identified increased allelic variation present in the three homoeoloci of the *SSIIa *gene by analyzing a mutagenised population of the bread wheat cultivar Cadenza, using a combination of forward genetics and TILLING. More recently, Uauy *et al. *[19] using a modified TILLING approaches detected novel allelic variants of *SBEIIa *and *SBEIIb *genes in tetraploid and hexaploid wheats.

The most established method for the detection of DNA polymorphisms used in TILLING is a heteroduplex mismatch cleavage assay based on the endonuclease Cel1 [17]. An alternative technology, High Resolution Melting™(HRM), deriving from the combination of existing techniques of DNA melting analysis with a new generation of fluorescent dsDNA dyes [20] could also be used. This method is sensitive and specific for the detection of mutations in PCR products from genomic DNA and has recently been successfully applied in TILLING [21,22].

In this work TILLING has been used to target genes encoding SBEIIa enzymes with the aim of developing non-transgenic wheat genotypes characterized by high amylose content and novel starch functionality.

## Results

### Selection of optimal genomic regions for TILLING

TILLING in polyploid species is complicated by the requirement for homoeoallele specific PCR for optimal sensitivity in SNP detection. As the three *SBEIIa *homoeoalleles share high similarity in their coding sequences, the intronic regions of the three genes were compared to identify sequence polymorphisms to facilitate the design of allele specific PCR primers. PCR amplicons for TILLING were also chosen to fulfill certain conditions. As our main objective was to identify functional mutations in the targeted genes, the exon density of potential amplicons was evaluated in order to select fragments that were as rich as possible in coding sequence. A further criteria used for the selection of TILLING fragments was the probability of finding deleterious SNPs (mutations affecting splicing sites or introducing stop codons) considering the types of transition mutation generally induced by EMS treatment (G → A; C → T).

**Figure 1:**
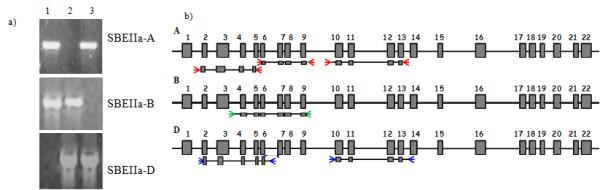
**Design and testing of primers for first round PCR**. **a) **Electrophoretic profile of the PCR products obtained from Langdon (1), Langdon 2D(2A) (2), Langdon 2D(2B) (3) by using homoeoallele specific primer pairs. **b) **Graphical representation of the first round PCR amplicons. For *SBEIIa-A *the selected regions are: fragment from exon II to V (A**^(II-V) ^**); from exon VI to IX (A^(**VI-IX**)^); from exon X to XIII (A^(**X-XIII**)^). For *SBEIIa-B*: from exon IV to IX (B**^(IV-IX)^**). For *SBEIIa-D*: from exon II to VI (D**^(II-VI^**)); from exon X to XIII (D^(**X-XIII**) ^). Red, green and blue arrows represent PCR primers specific for genome A, B and D, respectively.

Genomic regions selected for TILLING analysis are shown in Figure 1b. The amplicons vary in length between 1700 and 2200 bp. Three distinct regions of the gene were selected for the *SBEIIa-A *homoeoallele, two for *SBEIIa-D *and one for *SBEIIa-B*. Genome-specific primer pairs were designed for each target and validated for specificity using D-genome disomic substitution lines of the homoeologous group 2 chromosomes, produced in the durum wheat cultivar Langdon by Joppa and Williams [23] (Figure 1a).

### Detection of SNPs by HRM

The EMS-mutagenized population of bread wheat has been described elsewhere [24]. Briefly, this was derived from seeds of the UK spring wheat cultivar Cadenza treated with either 0.6% or 0.9% EMS solution overnight followed by growth to maturity. Single ears were harvested from each of the M_1 _plants and one grain from each ear sown to generate an M_2 _population of ~4,500 unique lines. Genomic DNA was isolated from the leaves of individual M_2 _plants and M_3 _seeds were harvested and archived. The M_2 _DNA samples were pooled two-fold and screened for mutations in the targeted regions (A**^(II-V)^**, A^(**VI-IX**)^, A^(**X-XIII**)^; B**^(IV-IX^**); D**^(II-VI^**) e D^(**X-XIII**) ^of *SBEIIa *(Figure 1b).

HRM was selected as the most suitable method for the detection of SNPs in the target genes considering their peculiar genomic structure. *SBEIIa *genes each contain 22 exons with sizes ranging between 40 bp and 240 bp spanning a region of 10 kb; moreover each exon is separated by introns of up to 1 kbp in size. In order to limit the number of mutations detected in introns and noting that HRM is most sensitive for the analysis of smaller fragments (100-400 bp), we chose to produce amplicons for HRM each covering the region of a single exon. As it was difficult to design homoeoallele-specific primers for each exon, amplicons with optimal sizes for HRM analysis were produced by nested PCR. First round, homoeoallele specific PCR fragments, as described above, were used as templates in 2^nd ^round PCR using primer pairs targeting each included exon. The 2^nd ^round primers were designed in the introns flanking each target exon and positioned approximately 5-20 nucleotides from the splice sites, resulting in PCR amplicons for HRM ranging in size from 100 bp to 350 bp.

### Optimization of HRM analysis

The principle of the HRM technique is based on the change in fluorescence of a dsDNA-specific intercalating dye during temperature-induced denaturation of the DNA duplex. The HRM instrument allows the monitoring of fluorescence changes in real time as the temperature of the samples is slowly increased. While detection of SNPs in homoduplex DNA is possible, instability created by the presence of mismatched bases in heteroduplex DNA increases sensitivity, producing a melt curve usually characterized by a loss of fluorescence at a lower temperature than wild type homoduplex DNA [20]. For TILLING assays, heteroduplexes are derived from the melting and re-annealing of wild type and mutant amplicons, generated by two-fold pooling of genomic samples before PCR.

**Figure 2:**
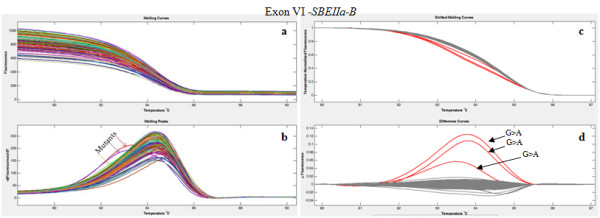
**High Resolution Melting analysis of second round PCR products of 96 2-fold pooled samples**. The figure shows the analysis of the amplicon correspondent to exon VI of the *SBEIIa-B *gene. **a) **Total fluorescence (F) vs temperature (T) curves; **b) **comparison of dF/T curves between normal and heteroduplex (indicated by arrows) DNA amplicons; **c) **normalized and temperature-shifted curves of fluorescence vs temperature showing wild types (grey) and mutants (red); **d) **ΔF/T difference curves with variants highlighted in red.

For each second round primer pair, optimization of the conditions for PCR and the subsequent HRM step were carried out, noting that the presence of the LCgreen Plus dye increased the primer Tm and thus raised the optimum annealing temperature of the PCR reaction. Analysis of the melt curve of the amplicon also allowed the specificity of the PCR to be confirmed. Although the presence of the mutation has been detected comparing the ΔF/T curves (Figure 2, panel d), produced by the HRM software, the observation of dF/dT curves (Figure 2, panel b) has proved useful for further confirmation of the mutations. In fact, heteroduplexes show a dF/dT curve visibly shifted at lower temperature in comparison with normal amplicons. All the amplicons have been analyzed in the temperature range between 75°C- 95°C; the two amplicons covering exon II and exon V have been further analyzed at higher temperatures to optimize the analysis of their GC rich domains (data not shown).

### Novel allelic variants for *SBEIIa-A*, *SBEIIa*-*B *and *SBEIIa*-*D *homoeoalleles

Screening of genomic DNA from the TILLING library was conducted on two fold pools in consideration of the high mutation density associated with this hexaploid wheat EMS-mutagenised population. In Table 1 the numbers of plants analyzed and mutants identified for each of the three genes *SBEIIa-A, SBEIIa-B *and *SBEIIa-D *are reported. The mutation density has been calculated as follows: (total size of amplicons) × (total number of screened lines)/(number of identified mutations). Of the 53 novel alleles (plus three duplicated mutations) of *SBEIIa*-*A *that were characterized, 36 were mis-sense, 15 silent and two truncation mutations. 50 novel alleles (plus three duplicated mutations) were identified for the *SBEIIa*-*D *gene of which 34 were mis-sense, 14 silent, 1 on the splice junction and 1 non sense mutation. Of the 14 novel *SBEIIa*-B alleles 10 were mis-sense, 1 truncation and 1 splice junction mutation (Table 2, 3). The 18 putative mutants identified in the amplicon A^(X-XIII) ^were not characterized by sequencing with the exception of one nonsense allele localized in exon XII.

**Table 1:** Overview of TILLING analysis.

Amplicon	Size (bp)	N° Plants analyzed	Mutations	Mutations density (kb per mutation)
A^(**II-V**)^	493	2300	30	39
A^(**VI-IX**)^	358	2688	26	40
A^(**X-XIII**)^	498	1531	18*	34*
B^(**IV-IX**)^	500	1152	14	40
D^(**II-VI**)^	580	1920	23	31
D^(**X-XII**)^	498	1920	30	33

*Mutations in amplicon A^(**X-XIII**) ^have not been characterized by sequencing with the exception of non sense mutation C2907T in exon X.

**Table 2:** Description of the mutations detected by TILLING.

Gene	Non coding	Silent	Missense	Nonsense	Splice Junction
*SBEIIa-A*	3	15	36	2	0
*SBEIIa-B*	0	2	10	1	1
*SBEIIa-D*	3	14	34	1	1

**Table 3:** Mutations affecting enzyme functionality as predicted by PARSE-SNP application.

Gene	Nucleotide change	Mutation effect	**PSMM diff**.
	G483A	G66D	32.06
*SBEIIa-A*	G485A	E67K	10.05
	C1748T	P206S	16.09
	C1755T	A208V	10.09
	C2907T	Q301*	
	G5165A	W436*	

	G1916A	S. J.	
*SBEIIa-B*	C1765T	A205V	14.06
	G1948A	W220*	
	G511A	G62S	32.01
	G520A	D65N	15.05

	G1774A	A201T	13.08
*SBEIIa-D*	C3693T	Q346*	
	C3916T	H362Y	18.04
	G3952A	G374R	22.08
	G4000A	G390S	14.01
	G4024A	V398I	14.07
	G5278A	D462N	28.06
	G5335A	S. J.	

The symbol "*" indicates nonsense mutations. S. J.= Splice Junction.

We estimated an overall mutation density of 1 mutation per 40 kb screened. All mutations identified were shown to be transitions of the type C→T or G→A as expected for treatment with EMS, which acts via alkylation of G residues. The knock-out genotypes (C2907T and G5158A) identified for *SBEIIa-A *allele, respectively in exon IX and XII, will be referred to as *SBEIIa-A^-1 ^*and *SBEIIa-A^-2^*; the two null genotypes for *SBEIIa-B *are named as *SBEIIa-B^-1 ^*(G1948A, non sense mutation in exon VI) and *SBEIIa-B^-2 ^*(G1916A, 3' splice site of intron V); the mutants C3693T (non sense mutation in exon X) and G5335A (5' splice site of intron XIII) of D genome allele are respectively named *SBEIIa-D^-1 ^*and *SBEIIa-D^-2^*.

**Figure 3:**
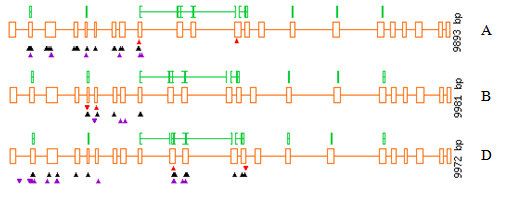
**Representation of the allelic variants identified in *SBEIIa *genes by TILLING as obtained by PARSESNP**. Red, black and violet triangles represent deleterious (non-sense and splicing junction), mis-sense and silent mutations, respectively.

Non-synonymous SNPs result in an amino acid change in the protein that can affect protein functionality to varying extents. In order to evaluate the effect of mis-sense mutations identified, the web based program PARSESNP http://www.proweb.org/parsesnp/ has been used (Table 3; Figure 3). PARSESNP utilizes two different bioinformatic tools, PSMM (Position-Specific Scoring Matrix) and SIFT (Sorting Intolerant from Tolerant ) which predict whether an amino acid substitution affects protein function based on sequence homology and the physical properties of amino acids [25]. PARSESNP analysis of the non-synonymous mutations found in *SBEIIa-A*, *SBEIIa-B *and *SBEIIa-D *resulted in the identification of 4, 1 and 8 mis-sense mutations, respectively, that are predicted to have severe effects on protein functionality. For the four protein variants SBEIIa-A ^(P206S)^, SBEIIa-A ^(A208V)^, SBEIIa-B^(A205V) ^and SBEIIa-D^(A201T) ^the amino acid change induced by the EMS treatment is located in the region of the N-terminal domain of the glycogen branching enzyme family, reported to be essential for the size of the glucan chains transferred and also for the catalytic activity of BE [26]. The amino acids changes H362Y, G374R, G390S, V398I and D462N, identified for the SBEIIa-D protein, are all localized in the (α/β)_8 _barrel catalytic domain of related enzymes belonging to the α-amylase family. Secondary structures and catalytic residues were identified in the three SBEIIa proteins through homology with the crystallographic structure of glycogen branching enzyme of *E. coli*, the model protein for branching enzyme family [27]. On the basis of these information it has been determined that the amino acid changes G390S and D462N are localized in the two strands β3 and β4 respectively of the (α/β)_8 _barrel domain; H362Y is adjacent to the residue Tyr361 known to be involved in catalysis, while V398I is located between Asp396 and His401 also directly involved in enzymatic activity.

**Figure 4:**
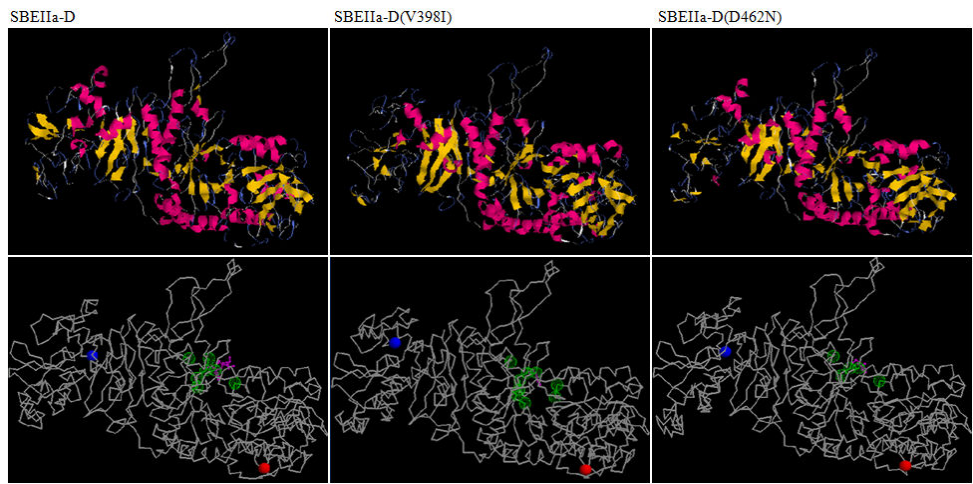
**3D Structures of normal and mutated SBEIIa-D protein**. Secondary (above) and 3D (bottom) structures as elaborated by I-TASSER for wild type and mutant forms of SBEIIa-D protein (V398I and D462N). The ligand is depicted in magenta colored ball & stick, the predicted binding site residues interacting with the ligand are shown as transparent green spheres, while the N and C terminus in the model are marked by blue and red spheres respectively.

In order to study more in detail the new SBEIIa variants described above, the amino acid sequences were submitted to the program i-Tasser http://zhanglab.ccmb.med.umich.edu/I-TASSER/[28] which predicts the 3D structures and functionality of the proteins. The comparison between the simulated 3D structures of non mutated and mutated SBEIIa proteins, in most cases, highlighted differences in the pattern of substrate binding sites and in the protein secondary structure. In Figure 4 we show as an example the case of SBEIIa-D^(V398I)^: while in wild type protein residue 398 was involved in the β3 strand of the (α/β)_8 _domain, in the mutated protein it is in a coil structure. Moreover the program predicted a different pattern of substrate binding sites for normal and mutated protein: of the seven binding sites predicted for the normal SBEIIa-D, in SBEIIa-D^(V398I) ^six residues were conserved and two new residues resulted involved in substrate binding (Figure 4). On the contrary in SBEIIa-D^(D462N) ^the mutation caused the loss of two of the seven amino acids involved in the binding and catalytic activity in normal SBEIIa protein, respectively Arg465 and Asp467.

### Analysis of *SBEIIa*-transcripts in the knock out mutants

Expression of the three *SBEIIa *genes was evaluated in homozygous lines of the five putative knock out mutants, *SBEIIa*-*A^-1^*, *SBEIIa*-*A^-2^, SBEIIa*-*B^-1^*, *SBEIIa*-*B^-2 ^*and *SBEIIa*-*D^-1^*. All of these alleles are non-sense mutants with the exception of *SBEIIa-B^-2^*, which is a splice-site mutation. Allele-specific qRT-PCR primer pairs were designed by comparing coding regions of the three *SBEIIa *genes. In some cases specificity was provided by the presence of small indels between the three genes; otherwise primers were designed based on sequence polymorphism in their 3' terminal ends. The specificity of the primers was validated by PCR on genomic DNA of the Langdon D-genome disomic substitution lines. Semi-quantitative and real time qRT-PCR experiments were performed on total RNA isolated from immature seeds (18 dpa) of homozygous mutant lines to investigate whether the expression levels of *SBEIIa *genes were affected by the presence of the putative knock-out mutations in the *SBEIIa *single null genotypes.

**Figure 5:**
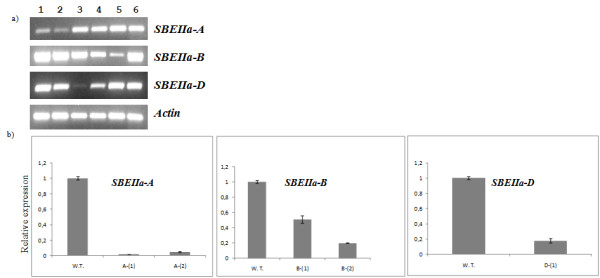
**Semiquantitative and quantitative RT-PCR of *SBEIIa *transcripts**. **a) **Semiquantitative RT-PCR of *SBEIIa *genes in *SBEIIa *homozygous single mutant genotypes: 1) *SBEIIa-A^-1^*; 2) *SBEIIa-A^-2^*; 3) *SBEIIa-D^-1^*; 4) *SBEIIa-B^-1^*; 5) *SBEIIa-B^-2^*; 6) wild-type Cadenza. **b) **Relative expression of *SBEIIa *homoeologs in single null genotypes as determined by Real Time quantitative PCR analysis: W.T.= wild type Cadenza; A-(1)= SBEIIa-A^-1^; A-(2)= SBEIIa-A^-2^; B-(1)= SBEIIa-B^-1^; B-(2)= SBEIIa-B^-2^; D-(1)= SBEIIa-D^-1^. Vertical bars indicate standard error.

Figure 5a clearly shows a drastic decrease of *SBEIIa-A *transcript in the two non-sense mutant lines *SBEIIa-A^-1 ^*and *SBEIIa-A^-2 ^*compared to the wild type genotype. A similar effect was found in the *SBEIIa-B^-2 ^*and *SBEIIa-D^-1 ^*genotypes, showing a severe reduction in transcript level due to both the splicing and non sense mutations, respectively, on the expression of the genes. In one case, *SBEIIa-B^-1^*, the presence of premature stop codon in the gene sequence has not resulted in a strong reduction of its transcript. Each mutant genotype was also investigated for the expression of the two remaining wild-type homoeologous copies of *SBEIIa*. No appreciable difference was detected in this case with respect to the wild type plant.

The extent of gene silencing in the five putative knock out mutants was quantified by Real Time RT-PCR (Figure 5b). We registered the strongest effect on gene expression in the two *SBEIIa-A *null lines, *SBEIIa-A^-1 ^*and *SBEIIa-A^-2^*: transcripts of the target alleles were found to be reduced to 1.7% and 3.3%, respectively, of the level in the wild-type control. Weaker effects were identified in the other null genotypes: the B alleles, *SBEIIa-B^-1 ^*(non-sense) and *SBEIIa-B^-2 ^*(splice site), were found to be expressed at 20% and 12%, respectively, of wild-type levels and *SBEIIa-D *allele was found 8.5 fold reduced in the *SBEIIa-D^-1 ^*genotype.

In order to investigate the effect of splice junction (S.J.) mutation (3' S.J. of intron V) on gene transcription, primers spanning exons II to IX were used to isolate transcripts from the *SBEIIa-B^-2 ^*mutant. PCR amplification resulted in two bands of different size: the larger product showed the inclusion of the intron V, whereas the smaller one was found to contain a deletion of the first seven nucleotides of exon VI. The presence of the intron V in the longer transcript showed that mutation at 3' splice site of intron V caused an incorrect splicing of *SBEIIa-B*. The deletion in exon VI, found in the shorter fragment, is probably due to the selection of an alternative splice junction site, positioned 5 nucleotides downstream the normal S.J. site. This last mechanism has been previously found in plants [29,30] and explained by the local scanning of the spliceosome that may select the best intron 3' splice site on the basis of sequence context [31]. Splicing of the immature mRNA at this junction would result in a frame-shift mutation leading to the production of a premature stop codon.

### Estimation of amylose content, total starch and seed weight

In order to detect the phenotypic effect of null mutations in *SBEIIa *genes, amylose content was measured in the three single mutants *SBEIIa*-*A^-1^, SBEIIa*-*B^-1 ^*and *SBEIIa*-*D^-1 ^*(Table 4). Our results showed an increase of amylose content in the three genotypes between 6% and 12% in respect to the normal genotype.

**Table 4:** Seed weight and amylose content in *SBEIIa *single null mutants and in wild type plants.

Genotype	100 grain weight	Amylose content*	Total starch
Cadenza	3.3 ± 0.03	33.2 ± 0.22	59.5 ± 0.06
*SBEIIa-A^-1^*	3.0 ± 0.06	37.5 ± 0.46	55.1 ± 1.06
*SBEIIa-B^-1^*	3.2 ± 0.06	35.2 ± 0.33	56.2 ± 0,96
*SBEIIa-D^-1^*	3.2 ± 0.09	37.1 ± 0.36	56.6 ± 1.01
*SBEIIa*-*A^-1^B^-1^*	3.2 ± 0.05	39.4 ± 0.39	55.2 ± 0.03
*SBEIIa*-*A^-1^D^-1^*	3.1 ± 0.06	38.6 ± 0.4	54.7 ± 0.29
*SBEIIa*-*B^-1^D^-1^*	3.0 ± 0.02	39.9 ± 0.39	54.0 ± 0.23

Standard error is also reported. (*) Mean of six replicates

Double null lines *SBEIIa *(*SBEIIa*-*A^-1^B^-1^, SBEIIa*-*A^-1^D^-1^, SBEIIa*-*B^-1^D^-1^*) have been produced by crossing single null genotypes and selecting the F_2 _progeny as described in Material and Methods. Pyramiding of two null homoeoalleles results correlated with an increase in amylose content included between 17%- 21% compared to the wild type (Table 4). In addition, comparison of 100 seed weights did not highlight significant differences among the single and double null genotypes compared to the control, although total starch content resulted decreased between 2% and 8% in the single and double null genotypes (Table 4).

## Discussion

In the last twenty years, modification of starch has been highlighted by food scientists as a primary target to confer added value on cereal products for both nutritional and industrial uses [7]. Naturally occurring variation has been exploited in wheat to generate starch with novel properties [8,32]. In polyploids the effect of mutations in single homoeologues is often masked by inherent genetic redundancy; therefore forward genetic screening for mutations requires extensive screening based on effective isoenzymatic or molecular markers. In addition, the shortage of mutations for most target loci in natural population makes the identification of the desired genotypes a slow process [32]. Both for Waxy and SGP-1, the availability of assays able to distinguish the individual protein products of the three homoeologous genes led to the identification of complete sets of single null mutants that were used to alter starch functionality in wheat [10,32,33]. However, a negative aspect of breeding programs based on natural genetic variation is the phenomena known as linkage drag. Extensive backcrossing is therefore required to remove undesirable characters inherited from exotic parental material making the breeding program time consuming.

In this work TILLING has been employed as a tool to identify novel genetic variability in the *SBEIIa *loci. In TILLING the desired variability is generated within a commercial variety selected by the breeder or researcher thus reducing genetic drag, although backcrossing is still required to remove excess mutations that may affect other characters. One disadvantage of TILLING in polyploid crops, compared to other reverse genetics approaches such as RNAi, is the need to combine mutations in all functional copies of the gene encoding the target protein. Pyramiding of the three *null *alleles is currently being carried out including backcrosses with Cadenza and we aim to complete this task within two years. On the other hand, mutants identified by TILLING are not considered to involve genetic manipulation and are relatively free of public and legislative concerns and, unlike RNAi which requires the production of transgenic plants, can be immediately introduced into breeding programs and tested in the field. If in diploid species chemical mutagenesis gives the opportunity to easily detect phenotypic changes linked to mutations in key genes, polyploids possess a higher tolerance of mutations resulting in a higher density in the population. This offers the possibility of identifying a wide variety of mutations in the target genes by screening a realistic number of mutagenised individuals.

TILLING in *SBEIIa *genes resulted in the production of large allelic series representing a valuable resource not only for starch modification but also to study structure-function relationship in the targeted enzyme. SBEs are found to contain three domains: an amino-terminal domain, a carboxyl-terminal domain and a central catalytic domain [27,34]. The N-terminal region is important for specifying the chain length and is required for maximum enzyme activity [26,35]. In this work protein variants characterized by mutations in functional domains of SBE enzyme have been identified and analyzed by bioinformatic tools able to predict the effect of the amino acid substitution on protein structure and functionality.

Although several mis-sense mutations have been found that potentially affect enzyme activity, the polyploidy nature of wheat prevents the immediate assessment of those allelic variants on phenotype. Thus, in a crop breeding perspective, the mutations of interest are those one known to prevent complete gene expression such as non-sense and splicing site located polymorphisms. To increase the frequency of the detection of knock-out mutants, a careful selection of gene regions rich in codons CAA, TGG, CAG and CGA was performed. The CODDLE application http://www.proweb.org/coddle/ is useful to evaluate truncation mutations frequency in the gene sequence; however we found that a more accurate selection of the fragments can be performed by manual sequence analysis. Moreover we finally selected gene fragments whose size is larger than that limited by CODDLE (up to1500 bp).

In general an efficient detection of SNPs in a gene is dependent upon the production of specific PCR products thus requiring the development of homoeoallele specific primers. In wheat obtaining full sequence data for target genes can be a significant challenge, although this is likely to be eased considerably in the next few years as shotgun and fully assembled sequence data is made available. We were able to design homoeoallele-specific primer pairs by identifying polymorphisms that exist among the three *SBEIIa *genes. In some cases oligonucleotides were designed corresponding to *indel *polymorphisms; however, it was also possible to develop specific primer pairs using a 3' terminal SNP in both the forward and reverse primers. Alternatively, a recent work suggests that it may be possible to use non-homoeoallele specific PCR to detect mutation in polyploids [21], although in our hands this resulted in reduced sensitivity.

High Resolution Melting has been recently applied to TILLING in plant species including tomato and wheat [21,22,36]. It is a closed tube PCR-based assay requiring no further processing of PCR amplicons; this results in significant advantages both in terms of costs and time saving in respect to other TILLING methods such as Cel1 digestion [37]. In our work the choice of HRM was strongly suggested by the consideration of the structure of *SBEIIa *genes, which contain many small exons (43-242 bp) interrupted by sizeable introns. As HRM is most suitable for the analysis of fragments up to 400 bp [38], this allowed us to target individual exons within the *SBEIIa *genes. Although traditional TILLING, based on Cel1 digestion, permits the analysis of larger amplicons (up to 1500 bp), this has as consequence the detection of mutations in the intronic regions that, excluding those in intron splice sites, do not impact on protein function [18].

HRM permitted an efficient detection of SNPs in two-fold pools of genomic DNA. The high mutation frequency of the wheat population used in the present work did not require deep pooling to increase the throughput of the screening. Our finding of a mutation density of 1 SNP for each 40 kb is in agreement with a previous report [36] that cited similar results for the same wheat population screened by traditional Cel1-based TILLING.

Hofinger *et al. *[37] have recently reported that HRM is less efficient in the detection of mutations localized at a distance of less than 20 nt from the PCR primers. Our data are in agreement with this hypothesis; in fact in some cases PCR primers were designed at a distance of less than 10 nucleotides from 5' and 3' ends of the exons as suggested by HRM software for primer design supplied by the manufacturer and this condition could have limited the number of mutations detected in the splicing sites of the exons analyzed. Suggestive of this we detected only two mutation in the splicing sites and in both cases primers had been designed at a distance of at least 20 nt from the ends of the exons.

The four non-sense genotypes *SBEIIa-A^-1^, SBEIIa-A^-2^, SBEIIa-B^-1 ^and SBEIIa-D^-1 ^*present a premature stop codon localized in the first twelve exons of the *SBEIIa *genes that prevents the production of a protein containing a functional (α/β)_8 _barrel catalytic domain essential for the enzyme activity. Also the two genotypes *SBEIIa-B^-2 ^*and *SBEIIa-D^-2 ^*present splice junction mutations, respectively localized at 5' end of exon VI and at 3' end of exon XIII, that would prevent a correct translation of the catalytic domain of SBEIIa enzyme by the introduction of premature stop codons.

The study of the effect of non-sense mutations on gene expression in plants is a poorly-explored topic [39,40]. We found that non-sense mutations in the gene sequence were associated with a detectable decrease in transcript levels in respect to the control genotype. Moreover the splicing junction mutation in *SBEIIa-B^-2 ^*also has been associated to a significant reduction of the gene expression. For each mutant genotype we tested the expression level of all the three homoeologous *SBEIIa *copies finding that just the gene with non sense mutation (or mutation in the splicing site) presented drastic decrease in the level of expression. Saito and Nakamura [41] reported similar results for a *Wx-A1^- ^*mutant characterized by a premature stop codon in the gene sequence. Patron *et al. *[42] reported the characterization of a barley waxy mutant, derived by mutagenesis, in which a premature stop codon was associated to the absence of the protein product; in this case the transcript level of the mutant allele was found similar to that of wild type. Similar results were found by Zhu *et al. *[43] for the wheat mutant, obtained by chemical mutagenesis, lacking the high molecular weight glutenin subunit Bx14 due to the presence of a premature stop codon. The reduction of transcript level detected in our knockout mutants suggests an intervention of a mechanism of quality control preventing accumulation of non functional or deleterious truncated protein, which has been described previously and is known as Nonsense Mediated mRNA Decay (NMD) [44]. Although this mechanism has been extensively characterized in mammals, little is known about its mode of action in plants. NMD in mammals takes place in intron-containing genes when the premature stop codon is positioned 55 nucleotides or more upstream of the last exon-exon junction [45]. In plants NMD has been reported to act also in case of intronless genes [46] thus showing that different rules govern this mechanism in respect to mammals; however several genes containing a premature stop codon positioned 55 nucleotides upstream of the last exon-exon junction have been reported to be subjected to NMD in plants [41,47-49].

All our knock out mutant genotypes present the premature stop codon at 55 nucleotides upstream of the last exon-exon junction thus following the consensus of NMD in mammals. Although reduction in transcript levels of the mutated genes has been detected in all our genotypes, the extent of the decrement varied among the 5 genotypes. In particular the mutant *SBEIIa*-B^-1 ^did not show drastic decrease in transcript level of the mutated allele. Similar examples have been reported in literature [42,43] indicating that NMD is a complex mechanism and further elucidation is needed to understand its mode of action in plants.

Amylose content was estimated in the control, the three non sense genotypes, for which seeds were available and double null mutants derived from their crossing. The modest increase of amylose content in single null mutants is presumable due to the compensation exerted by gene redundancy in polyploids, similarly to what reported by Miura and Sugawara [50] and Konik-Rose *et al. *[51] for other genes involved in starch biosynthesis. Further increase in amylose content was also observed for the three double null lines obtained from the cross of the three single null mutants. In addition, our results showed a modest decrease in starch content in the set of single and double null *SBEIIa *genotypes not correlated to a loss of seed weight. The discrepancy could be due to the limitation of the method to estimate total starch in high amylose cereals as reported by McClearly *et al. *[52].

Concluding, as previously found for the other genes controlling amylose content in wheat [10,53], it has to be expected a much higher increment of amylose content in triple null *SBEIIa *wheat.

## Conclusions

Novel allelic variants have been identified for the three *SBEIIa *homoeologs in bread wheat that represent a valuable resource both for functional genomics studies and for wheat improvement. In particular a complete set of single null *SBEIIa *wheat lines have been identified and characterized both at molecular and phenotypic level. Genic expression of null alleles resulted deeply reduced showing the intervention of NMD mechanism to prevent the production of a non functional protein. The set of the three single and double null genotypes showed an increase in amylose content which can further be increased when triple null lines will be available. The complete null lines will be used in breeding activities aimed to increase the level of resistant starch in wheat end products.

## Methods

### Plant material

Production of the EMS-mutagenised population of the spring bread wheat cv Cadenza has been described previously [9,24].

### Primer design

Alignment of the three gene sequences were performed by ClustalW http://www.ebi.ac.uk/clustalw. Gene- and homoeoallele-specific primers for TILLING were designed using the PRIMER 3 program. PCR primers for TILLING analysis were validated using D-genome disomic substitution lines of homoeologous group 2 chromosomes of the durum wheat cultivar Langdon [23]. Genomic DNA was extracted from 0.2 g of green tissue as reported in Tai and Tanksley [54]. Primers pairs are reported in Table 5.

**Table 5:** Set of genome specific primer pairs used to produce TILLING 1^th ^PCR amplicons.

Amplicon	Oligo-forward (5'-3')	Oligo-reverse (5'-3')	T. annealing	Size (bp)
A^(II-V)^	cgctcgctcgctccaatc	gcaactggtcagtattcagtaagctaag	65°C	1720
A^(VI-IX)^	tctgagaatatgctgggacgtag	gttcgaaaatgctacatgctca	62°C	1560
A^(X-XIII)^	ccagtggtcagaatgcatcaac	gggaactatctaagactccgtagcac	67°C	2100
B^(IV-IX)^	atgtggtggatgggttatgg	tccatagaataaaccatcagaccg	62°C	1970
D^(II-VI)^	atcgcgcttcctgaacctg	gggctgaagcttaagacactgac	65°C	1980
D^(X-XIII)^	gaggcagtgggcatgtgaaagtc	ctagggaactatctaagactccgtagcac	67°C	2200

PCR reactions for primer evaluation were carried out in 50 μl final volume using 50-100 ng of genomic DNA, 1× Red Taq ReadyMix PCR reaction mix (1.5 U Taq DNA Polymerase, 10 mM Tris-HCl, 50 mM KCl, 1.5 mM MgCl2, 0.001% gelatine, 0.2 mM dNTPs) and 0.5 μM of each of the two primers. Amplification conditions for testing primers included an initial denaturation step at 94°C for 5 min, followed by 35 cycles at 94°C for 1 min, 62-67°C for 1 min and 72°C for 1 min, followed by a final incubation at 72°C for 5 min.

### Screening of the TILLING library

Amplicons analyzed in TILLING were produced by a nested PCR strategy. 1^st ^round PCR was carried out in a 10 μl volume using 10 ng of two-fold pooled genomic wheat DNA, 5 μl of Hot Shot™Mastermix (Cadama Medical Ltd), 0.5 μM primers. The PCR program was: 97°C, 5 min; (97°C, 30 s; 62-67, 30 s; 72°C for 1.5-2 min)x 38 cycles; 72°C, 10 min. 96 well plates were used for the screening.

For HRM, the 1^st ^round PCR reaction was diluted 60 fold and 1 μl was used as template in the 2^nd ^round PCR. The 2^nd ^round PCR reaction was prepared as follows: 1 μl of diluted DNA template (1:60); 5 μl of Hot Shot™Mastermix (Cadama Medical Ltd); 1 μl of LCGreen Plus; 0.5 μM primers (Table 6). The PCR program used was: 97°C, 5 min; (97°C, 30 s; 60°C, 20 s; 72°C, 20-30 s)x 39 cycles; 72°C, 10 min. After the final extension step, PCR amplicons were denatured at 95°C for 30 s and reannealed at 25°C for 1 min. Both 1^st ^and 2^nd ^round PCR reaction were overlaid with 10 μl of mineral oil (Sigma-Aldrich M5904) to prevent sample evaporation. 2^nd ^round PCRs were run in 96 well Frame-Star plates (4titude Ltd, Surrey, UK).

**Table 6:** Set of gene specific primer pairs used to produce TILLING 2^nd ^PCR amplicons.

Allele	Exon	primer forward (5'-3')	Primer Reverse (5'-3')	Size (bp)
	II	ccactgaccgactcact	atggacggggagattgg	215
	III	tcactattgtagtcatccttgcat	tgaagatttcccggcacg	156
	IV	tggtttcgttagtctgctct	tgagcgaaagtagcggg	313
	V	tttgggtatgcctccgt	tggaggcgcttcataatact	163
	VI	ttgctctaaatttatgatctggct	aggtggaagattgccaag	153
*SBEIIa-A*	VII	tgctcctattgatgccgat	gctacatgctcaactaaataattgg	152
	VIII	ctctgcccactaagggt	aaatttcatttaataatgtaatggagatcg	204
	IX	ccttttgtgaccatttactaaggata	accagaaacaggtgaaataact	157
	X	acaatacttagaggatgcatctga	ggtgaagaggcgcataca	212
	XI	ggtatttctgacttgtatgaccatt	accagataaacagtaaagcagc	223
	XII	gttgcattgcttcatcaatgatt	caaatatggtgacagaagtcagag	237
	XIII	tgttaaatctgttcttacacatgtcg	catagcaattatttcagtgccct	266

	IV	aacacactgctaaatttgaatgat	agactagtggaggcgtt	19
	V	tgctgaaggtatcatctaattgc	tgaccattaacaatagattagaaggtg	159
*SBEIIa-B*	VI	cagttactctaaatttatgatctggct	aggtggaagattgccaag	134
	VII	cctattgatgccgatatttgatatg	tcctcgactaaataattggccag	152
	VIII	aactctgcctactaagggt	acactggaaattccatttaataatgtaac	204
	IX	ccttttgtgaccatttactaaggata	ccggaaacaggtgaaataact	157

	II	actattgtagtcatccttgcatt	atgaagatttaccggcacg	157
	III	tcagtctgctctacaattgctat	gaaagcagcgggtaggc	301
	IV	gggtatacctcggtggattc	agactagtggaggcgttt	167
	V	gaaggtatcgtctaattgcatatct	caataaattggaaggtgtctcgtt	154
*SBEIIa-D*	IX	accatttactaaggatatttacatgcaa	accagaaacaggtgaaataact	151
	X	acaatacttagaggatgcatctga	ggtgaagaggcgcataca	212
	XI	ggtatttctgacttgtatgaccatt	accagataaacagtaaagcagc	223
	XII	gttgcattgcttcatcaatgatt	caaatatggtgacagaagtcagag	237
	XIII	tgttaaatctgttcttacacatgtcg	catagcaattatttcagtgccct	266

### High Resolution Melting by LightScanner

The 96 well plates (2^nd ^PCR) were used for HRM using the LightScanner instrument (Idaho Technology, Inc). Samples were normally heated using a temperature range from 75°C to 95°C. For amplicons containing high GC regions a further analysis was conducted in a temperature range from 85°C to 98°C to guarantee optimal resolution in SNP detection.

The data obtained were analyzed by LightScanner software analysis provided with the instrument. Melting curves were normalized according to the manufacturer's instructions. The results obtained by HRM were visualized as differential curves ΔF/T displaying the relative difference in fluorescence of a respective sample in respect to a reference sample. F/T normalized curves show the decrement in fluorescence of each sample during the denaturation of the PCR amplicon as the temperature increases. As stated by the manufacturer's instructions, ΔF > 0.05 was considered significant; furthermore the shape of the melting curve and position (along temperature axis) along the dF/T curve were observed and used as criteria to distinguish false positive from real mutants. Samples identified as putative mutants were selected and the amplicon re-amplified from each individual in the pool for sequencing. DNA sequence analyses were conducted by a commercial sequencing service (Eurofins MWG Operon, Ebersberg, Germany). The PARSESNP http://www.proweb.org/parsesnp/ application was used for the evaluation of protein variants coded by mutated alleles.

### Semi-quantitative reverse transcriptase-polymerase chain reaction

Total RNA was extracted from immature seeds (18 DPA) as reported in Laudencia-Chingcuanco *et al. *[55] with some modifications. The starting material was 0.1 g and all volumes of buffers and solutions were diluted 1 to 10. For reverse transcriptase-mediated PCR studies, cDNA was synthesized from 1 μg of total RNA using an oligo(dT) primer and Superscript Reverse Transcriptase III (Invitrogen). One of twentieth volume of each cDNA was used as a template for PCR amplification. PCR reactions were carried out in 20 μl final volume using 1 units of Ex-Taq (Takara), 1× buffer, 0.2 mM of each dNTPs, 0.5 μM of each primer. Amplification conditions included an initial denaturation step at 98°C, followed by 35 cycles at 98°C for 10 sec., 58°C for 1 min. and 72°C for 1 min, followed by a final extension at 72°C for 5 min. The following gene-specific primers were designed for: *SBEIIa*-*A *[EMBL:HE591389] (5'accagtatgtttcacggaaacac3'; 5'caccttgtacttcccaggcc3'), *SBEIIa*-*B *[EMBL: FM865435] (5'atatcgtggtatgcaagagttcgac3'; 5'caagaaagagcgcggccta3'), *SBEIIa*-*D *[GenBank: AF338431] (5'gaggaagataaggtgatcatcctca3'; 5'caaagagtgcatcgtcagagtcc3'). Amplification of the wheat actin gene [GenBank: AB181991] was used as reference for transcript amplification and the primers used have the following sequence: (TaACTINF) 5'-aagagtcggtgaaggggact-3' and (TaACTINR) 5-ttcatacagcaggcaagcac- 3'.

### Isolation of the *SBEIIa-B *mRNA sequences of the splice junction mutant

Gene transcripts were isolated from cDNA by using the homoeoallele specific primer 5'gacttggcggccactcca 3' and the gene specific primer 5'ctctggtcgtttaggttgaggatg 3'.

### Real-Time RT-PCR (qRT-PCR)

One microlitre of the cDNA prepared above was used for real-time PCR in a 20 μL volume. For each sample three technical replicates were used for PCR amplification. The PCR reaction consisted of 10 μL of iQ™ SYBR Green Supermix 2× (BIO-RAD), which contained buffer, dNTPs and SYBR Green I. Concentrations of the forward and reverse oligodeoxynucleotide primers in the reaction were 500 nM for all the genes of interest. qRT-PCR experiments were performed using the *iCycler iQ *(Bio-Rad Laboratories, Hercules, CA1, USA). Amplification conditions were as follows: initial 95°C for 15 min and 40 cycles of 95°C for 30 s, 60°C for 1 min and 72°C for 1 min each.

Relative expression analysis was determined by using the 2^-ΔΔCT ^method [56] (Applied Biosystems User Bulletin No. 2-P/N 4303859). Calculation and statistical analyses were performed by Gene Expression Macro™ Version 1.1 (Bio-Rad Laboratories, Hercules, CA, USA). The efficiencies of target and housekeeping genes were determined by qRT-PCR on serial dilutions of RNA template over a 100-fold range [57], with similar results (data not shown). Amplified products were checked by gel electrophoresis and sequencing to verify primer specificity. The relative expression of each gene is reported as the fold increase of the transcript level at each time point, compared to the lowest transcript level. As in semi-quantitative RT-PCR, actin was used as the housekeeping gene.

### Selection of double null *SBEIIa *mutants

Double null *SBEIIa *lines were obtained by crossing *SBEIIa-A^1- ^*, *SBEIIa-B1- *and *SBEIIa-D1*^-^. Double null homozygous lines of the F2 progenies were selected by PCR using CAPS or dCAPS primers followed by a specific restriction enzyme reaction. dCAPs primers have been designed by dCAPs Finder application [57]. Digested PCR amplicons were run on agarose gels (2%) stained with ethidium bromide for band visualization.

The following primer pairs and restriction enzymes were used: *SBEIIa-A^1- ^*(Fw taaatcctcagtgactctggtcgtttaggttgaggattc, Rv aagtgacatatgcattaattcaccttctaa; Xba); *SBEIIa-B^1^*- (Fw ctggagcgcatgtacgtcttaac, Rv caccataatcatcctgaaaagatcg; MfeI); *SBEIIa-D^1- ^*(Fw gaggcagtgggcatgtgaaagtc, Rv ccaaagcttgcatagtatgaatgctcctggattgccattgtcg; SalI)

### Determination of amylose and total starch content

Amylose content (percentage of total starch) was determined by a iodometric assay as reported in Chrastil [58] using starch extracted from whole flour by the "dough ball" method [59]. Seeds were obtained from plants grown in the field. Three biological and six technical replicates have been used for all materials.

A standard curve was used using mixtures of potato amylose (Fluka 10130) and amylopectin isolated from waxy wheat. Total starch content of kernels was determined by Megazymes Total Starch Assay Kit (AA/AMG, Megazyme Pty Ltd., Wicklow, Ireland).

## Authors' contributions

EB carried out the TILLING analysis, molecular and bioinformatic characterization of mutants and drafted the paper with FS and DL. AHL collaborated to the optimization of HRM analysis. AP provided the EMS wheat population and the HRM TILLING platform. EB, FS, AP and DL edited the manuscript. DL coordinated the work. All authors read and approved the final manuscript.
